# Effects of non-intubated anesthesia on postoperative pulmonary complications after thoracoscopic segmentectomy: a propensity score-matched analysis

**DOI:** 10.1080/07853890.2026.2721209

**Published:** 2026-08-26

**Authors:** Yawen Tan, Ge Liu, Di Wang, Yuan Zhang, Chuangang Li, Yunpeng Zhao, Lei Shan, Ning Li

**Affiliations:** ^a^ Department of Anesthesiology, The Second Qilu Hospital of Shandong University, Jinan, China; ^b^ Clinical Epidemiology Unit, Qilu Hospital of Shandong University, Jinan, China; ^c^ Department of Anesthesiology, Shandong Provincial Hospital Affiliated to Shandong First Medical University, Jinan, China; ^d^ Department of Thoracic surgery, The Second Qilu Hospital of Shandong University, Jinan, China

**Keywords:** Non-intubated anesthesia, postoperative pulmonary complications, thoracoscopic segmentectomy

## Abstract

**Background:**

Minimal invasive thoracoscopic segmentectomy is being increasingly performed in patients with early lung tumor. A combination of non-intubated anesthesia and thoracoscopic segmentectomy could be a less invasive procedure due to the avoidance of the double-lumen endobronchial intubation and mechanical ventilation. This study was aimed to explore the relationship between non-intubated anesthesia and postoperative pulmonary complications (PPCs) in thoracoscopic segmentectomy.

**Patients and methods:**

A retrospective propensity score matched study was conducted from November 2021 to July 2024 among patients who underwent non-intubated or intubated thoracoscopic segmentectomy from the same surgeon team. Propensity score matching was performed to establish 1:1 non-intubation versus intubation group matching to balance potential confounding factors. Primary outcome was the incidence of PPCs including respiratory infection, pneumothorax, atelectasis, pleural effusion, respiratory failure, bronchospasm, and aspiration pneumonitis, and was analysed by Chi-squared test (or Fisher exact test). PPCs severity grade was assessed as the second outcome, and analysed by Mann–Whitney *U* test.

**Results:**

After 1:1 propensity score matched, a matched cohort with 122 (61:61) patients was generated. The incidence of PPCs was lower in non-intubation group as compared to intubation group (16.4% vs. 37.7%; *p* = 0.008), wherein the reduction of respiratory infections was most significant (9.8% vs. 29.5%; *p* = 0.006). The severity score of PPCs was reduced in non-intubation group (*p* = 0.030).

**Conclusion:**

Non-intubated anesthesia was associated with lower PPCs incidence and milder severity grade in patients undergoing thoracoscopic segmentectomy.

## Key Messages

1. Non-intubated anesthesia could be a feasible and safe alternative to intubated anesthesia for selected patients undergoing thoracoscopic segmentectomy.
2. Non-intubated anesthesia is associated with significantly decreased incidence and severity of postoperative pulmonary complications (PPCs) in thoracoscopic segmentectomy.
3. Non-intubated anesthesia is related to shorter length of hospital stay and lower level of white blood cell after surgery.

## Introduction

Video-assisted thoracoscopic surgery (VATS) lobectomy is the gold standard surgery procedure for early-stage lung cancer [1]. Nevertheless, thoracoscopic anatomical segmentectomy has gradually been developed as an effective alternative procedure to VATS lobectomy for selected patients [2,3], which was superior to lobectomy for overall survival and noninferior for relapse-free survival in patients with small-sized peripheral non-small-cell lung cancer. As a lung parenchyma-preserving surgery, segmentectomy protected postoperative lung functions compared with lobectomy, which was beneficial to patients who cannot tolerate lobectomy and promoted the postoperative recovery [4,5]. However, postoperative pulmonary complications (PPCs) remain one of the most common complications in thoracoscopic segmentectomy, delaying patient recovery and increasing morbidity and mortality [6,7].

Traditional general anesthesia with double-lumen endobronchial tubes (DLTs) is the standard anesthesia method in VATS lung surgery and provides an adequate surgical environment through the implementation of one-lung ventilation (OLV) technique. However, invasive mechanical ventilation and the extensive use of anesthetics such as opioids and muscle relaxants could lead to PPCs [8–10]. Recently, non-intubated anesthesia has been safely implemented in VATS lung surgery and demonstrated advantages, including shorter chest-tube placement duration, shorter hospital stays and accelerated recovery processes [11–13]. Given the avoidance of intubated mechanical ventilation and significant reduction in opioids and muscle relaxants, non-intubated anesthesia may improve postoperative pulmonary function in patients undergoing VATS lung resection. Liu et al. reported that non-intubated anesthesia could reduce the incidence of respiratory complications in selected patients undergoing VATS lung surgery compared with intubated anesthesia [14]. However, there are few studies focused on the effect of non-intubated anesthesia on VATS segmentectomy. The relationship between non-intubated anesthesia and PPCs in VATS segmentectomy remains not clear.

In this study, a retrospective analysis was performed to explore the association between the PPCs and non-intubated anesthesia for patients undergoing VATS segmentectomy.

## Patients and methods

### Sample size estimation

According to a previous study [15] and our findings, the incidence of PPCs within 7 days following intubated anesthesia VATS segmentectomy was estimated at 40%. Based on our institutional historical data, we assumed a clinically relevant absolute reduction in PPCs to 16% (a 24% decrease) in patients undergoing non-intubated VATS segmentectomy. A sample size of 52 patients per group was required, with a two-sided *α* = 0.05 and *β* = 0.20. To compensate for the approximately 10% dropout rate during propensity score matching (PSM), we aimed to enroll at least 58 patients per group. Sample size estimation was performed with the PASS 15.0 software (NCSS Statistical Software, Utah, USA).

### Study design and patients

This single-center retrospective study was conducted in The Second Qilu Hospital of Shandong University. The study protocol was approved by the Clinical Research Ethics Committee of The Second Qilu Hospital of Shandong University (No. KYLL2025301) and [registered with the Chinese Clinical Trial Registry (www.chictr.org.cn, ChiCTR2500103544). The clinical information of patients undergoing non-intubated anesthesia or intubated anesthesia VATS segmentectomy were collected from inpatient medical record database between November 2021 and July 2024. Written informed consent was waived owing to the retrospective nature of this study. The study is in accordance with the Strengthening the Reporting of Observational Studies in Epidemiology (STROBE) checklist [16].

The inclusion criteria included (1) patients who underwent VATS segmentectomy; (2) age ranged from 18 to 80 years; (3) American Society of Anesthesiologists (ASA) physical status classification score of I to III. The exclusion criteria were as follows: (1) patients with difficult airway (Mallampati classification > III) or airway obstruction/obstructive sleep apnea–hypopnea syndrome; (2) patients with gastroesophageal reflux disease; (3) contraindications to nerve block, such as coagulopathy, local infection, allergy to relevant anesthetic; (4) history of lung resection; (5) history of acute upper respiratory tract infection within 1 week before surgery; (6) patients who contracted COVID-19 during hospitalization; (7) patients converted from non-intubated to intubated anesthesia intraoperatively.

### Anesthesia protocol and management

In the non-intubation group, a T5–T6 thoracic paravertebral block (0.5% ropivacaine, 10–15 mL) and cervical vagus nerve block (2% lidocaine, 3 mL) were performed under ultrasound before surgery. During anesthesia induction, dexmedetomidine (0.5 µg·kg^−1^) was infused intravenously within 15 min, and propofol was administered continuously at 4–6 mg·kg^−1^·h^−1^. When bispectral index (BIS) indicated adequate sedation (40–60), the laryngeal mask was inserted for oxygen supplement and carbon dioxide monitoring. The anesthetic dosages were adjusted to maintain a spontaneous respiratory rate between 10 and 15 cycles·min^−1^ and tidal volume at 4–6 mL·kg^−1^. During surgery, an incision of 3–4 cm in the fifth intercostal space and the anterior axillary line was administered after local infiltration anesthesia with 1% lidocaine. Once the pleural cavity was accessed, an open pneumothorax formed, which induced collapse of the lung. Then an additional 2% lidocaine (10 mL) was sprayed onto the pleura and multiple intercostal nerve blocks from T2–T9 (2 mL 0.5% ropivacaine per intercostal space) were applied under thoracoscopic guidance. Once the emergency situations such as severe hypoxemia (SpO_2_ <90%) or hypercapnia (pH <7.10) and significant mediastinal movement occurred and could not be controlled, a bronchial blocker would be inserted through the laryngeal mask or single-lumen tube under the guidance of fiberoptic bronchoscopy in the lateral position.

In the intubation group, the same paravertebral block was performed for analgesia. General anesthesia was induced with intravenous sufentanil (0.3 µg·kg^−1^), propofol (1.5–3.0 mg·kg^−1^), cisatracurium (0.2 mg·kg^−1^), and then DLT was inserted and located by fiberoptic bronchoscopy. Lung protective ventilation strategy was performed. The ventilator protocol comprised a tidal volume of 8 mL·kg^−1^ predicted body weight for two-lung ventilation and 6 mL·kg^−1^ for one-lung ventilation (OLV), PEEP of 3–8 cm H_2_O, an inspiratory-to-expiratory ratio of 1:2, and a plateau pressure limit <25 cm H_2_O. Respiratory rate was adjusted to sustain end-tidal carbon dioxide partial pressure (EtCO_2_) between 35 and 45 mmHg. Anesthesia was maintained with a continuous infusion of propofol (3–12 mg·kg^−1^·h^−1^) and remifentanil (0.1–0.3 μg·kg^−1^·h^−1^). When the surgery finished, the DLT was removed when the patient’s consciousness, muscle strength and breathing recovered well.

A standardized lung recruitment maneuver was applied in both groups, consisting of a continuous airway pressure of 30 cm H_2_O for 15–20 s at the restart of two‑lung ventilation. The fraction of inspired oxygen (FiO_2_) was initially set at 50% and then titrated incrementally up to 100% based on real-time oxygen saturation. Additionally, all surgical procedures were performed by the same surgical team and the restricted rehydration was administered for crystalloid fluid at 3–5 mL·kg^−1^·h^−1^. No urinary catheterization for all patients. After surgery the patients were observed in post anesthesia care unit.

### Data collection and outcomes assessment

The baseline information and perioperative data of all enrolled patients were collected using an electronic medical record system and telephone follow-up. The patients were divided into intubation group and non-intubation group according to whether performed DLT and mechanical ventilation.

Primary outcome was the incidence of PPCs which was recorded until hospital discharge but curtailed at postoperative day 7. The PPCs were defined according to the guidelines for European perioperative clinical outcome (EPCO) definitions, including respiratory infection, pneumothorax, atelectasis, pleural effusion, respiratory failure, bronchospasm, and aspiration pneumonitis (Supplemental Table 1) [17]. All patients underwent X-ray examinations before discharge. Pneumothorax and pleural effusion were considered as positive only when they occurred in the non-operative side. Researchers who assessed PPCs were blinded to group allocations.

Second outcomes included PPCs severity grade (Kroenke Score [18], Supplemental Table 2); the incidence of extrapulmonary complications within 7 days, defined as new non-respiratory events occurring postoperatively and classified as grade II or greater in the Clavien–Dindo classification (Supplemental Table 3) [19]; the incidence of postoperative pulmonary air leakage, defined as lasted air leakage for more than 7 days or requiring invasion treatment (adhesive agent intrathoracic injection or closed thoracic drainage); duration and volume of closed chest drainage; the length of postoperative hospital stay and postoperative day 2 serum inflammatory markers, including white blood cell (WBC), C-reactive protein (CRP), human neutrophil lipocalin (HNL), interleukin-6 (IL-6), and interleukin-10 (IL-10).

Safety outcomes encompassed intraoperative hypoxemia, hypercapnia, mediastinal movement graded by the classification system established in our previous research [20], cough interference, R0 resection rate, number of lymph nodes sampled or dissected, estimated blood loss, blood transfusion, intraoperative air leak, conversion to intubated anesthesia, conversion to thoracotomy, unplanned ICU admission, in-hospital mortality, reoperation, 30-day unplanned readmission and mortality.

### Propensity score matching

Based on clinical experience and published literature [21–23], propensity score matching (PSM) was performed to adjust for baseline characteristics, including age, sex, Body mass index (BMI), Forced expiratory volume in 1 s (FEV_1_) (%pred), FEV_1_/Forced vital capacity (FVC) (%pred), Assess Respiratory Risk in Surgical Patients in Catalonia (ARISCAT) score (Supplemental Table 4), smoking, ASA Physical Status Classification, Charlson Comorbidity Index, simple or complex segmentectomy stratified by operative procedure and intersegmental plane status [24], pathology (benign/malignant). All covariates were evaluated for multicollinearity using the variance inflation factor (VIF). A VIF threshold of <10 was considered acceptable. R 4.5.1 Package MatchIt was applied to perform nearest neighbor matching based on propensity scores calculated using a multivariable logistic regression model. The matching procedure was specified with random order and 1:1 ratio with caliper of 0.2. The balance of baseline variables was assessed using standardized mean differences (SMD), computed by R Package TableOne. SMD < 1.96×$\sqrt{\text{(}n1+n2)/(n1\times n2)}$ was considered acceptable balance [25].

### Statistics and analyses

The normality of continuous variables was evaluated using the Kolmogorov–Smirnov test. Data were summarised as counts and percentages, means and standard deviations (SD), or medians and interquartile ranges (IQR) as appropriate. Continuous variables were analyzed using the Student *t* test or Mann–Whitney *U* test, where applicable. Chi-squared test, continuity-corrected chi-squared or Fisher exact test was used for categorical variables; differences were expressed as relative risks (RRs) and 95% CIs. Multiple imputation by chained equations was performed using the R package mice (version 4.5.1). The imputation model included outcome variables (PPCs, PPCs severity grade, extrapulmonary complications, postoperative pulmonary air leakage, hospital stay), the exposure variable (intubation strategy), covariates (WBC, CRP and blood gas parameters), and patient characteristics (age, sex, BMI, FEV_1_, FEV_1_/FVC, ARISCAT score, smoking, ASA physical status, Charlson Comorbidity Index, segmentectomy, fluid volume, operation time, pathology). Imputation was conducted using predictive mean matching to generate 20 imputed datasets under the missing-at-random assumption. The imputed dataset with the smallest mean square error (MSE) was selected for subsequent analyses. R 4.5.1 and SPSS (version 17.0; SPSS Inc., IBM, Chicago, IL, USA) were used for data analyses. A two-sided *p* value of <0.05 was considered significant. In addition, Subgroup analysis was assessed using logistic regression, including sex, age, BMI, ARISCAT grade, type of pulmonary segmentectomy, operative duration, pathology.

The sensitivity analysis was prespecified. In order to obtain a more robust comparison, we employed the Melbourne Group Scale (MGS) [26] and the Standardised end-points in perioperative medicine (StEP) [27] to reevaluate PPCs in the matched cohort. Moreover, patients who were converted to intubated anesthesia or thoracotomy were maintained for intention-to-treat (ITT) analysis. Additionally, multivariate logistic regression analysis was performed to evaluate the independent association between non‑intubated anesthesia and PPCs in the ITT cohort. Univariate logistic analysis was firstly performed for baseline and intraoperative factors, including intubation strategy, age, sex, BMI, FEV_1_ (%pred), FEV_1_/FVC (% predicted), ARISCAT grade, smoking status, ASA classification, Charlson Comorbidity Index, segmentectomy type (simple vs. complex), pathology (benign vs. malignant), total fluid volume, one‑lung ventilation time, concurrent wedge resection, pleural adhesiolysis, systematic lymph node dissection or sampling, operative time, estimated blood loss, conversion to thoracotomy, and intraoperative air leak. Variables with *p* < 0.2 in univariate logistic analysis were integrated into multivariate logistic regression analysis. Backward stepwise regression analyses were used to identify independent factors.

## Results

### Patient characteristics

**Figure 1.**
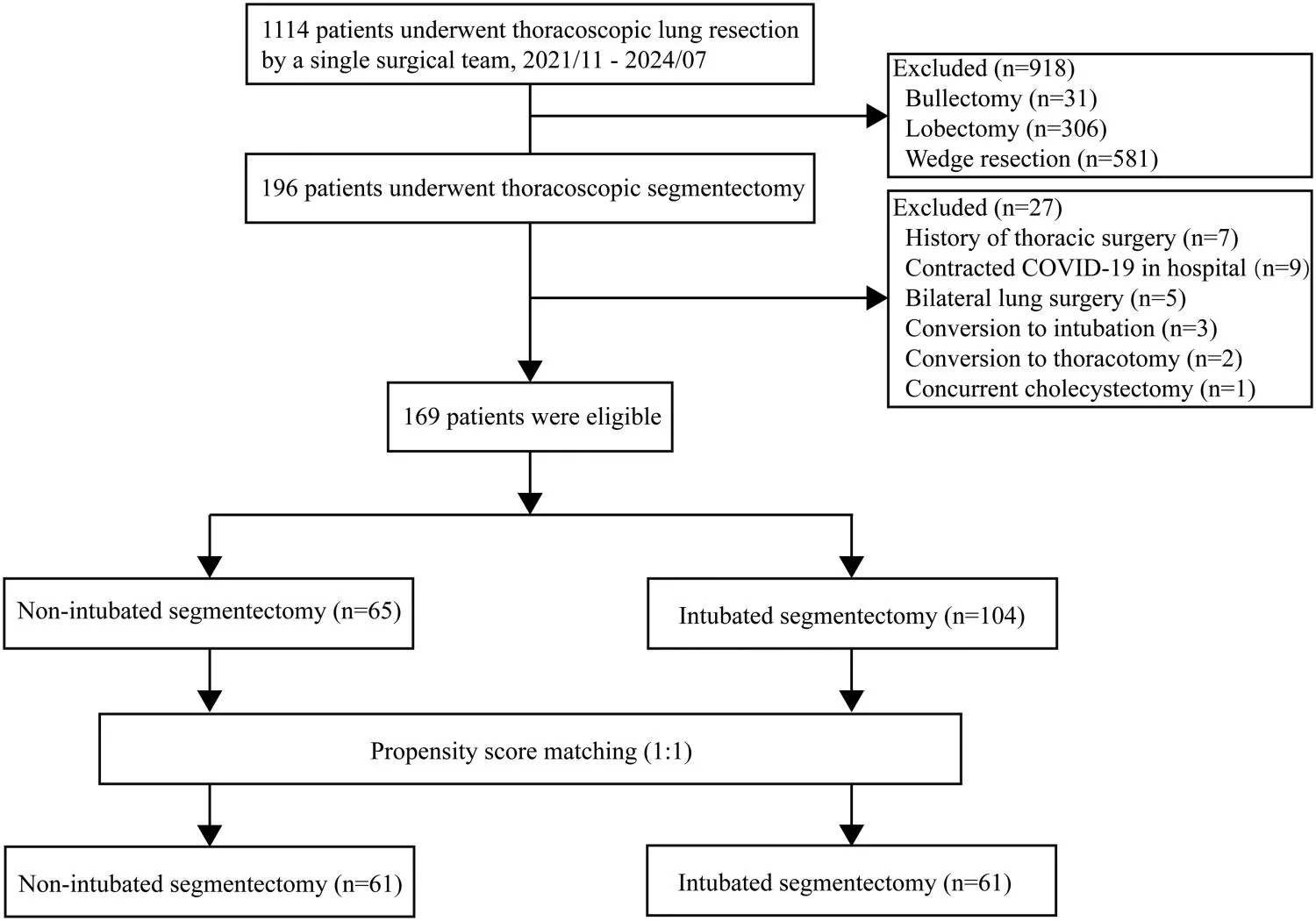
Study flowchart.

After exclusion of other types of thoracoscopic lung surgery, a total of 196 patients underwent elective VATS segmentectomy performed by the same surgical team from November 2021 to July 2024. Of these, 27 patients were excluded based on the exclusion criteria: 7 with history of thoracic surgery, 9 with hospital-acquired COVID-19, 5 undergoing bilateral procedures, 2 converted to thoracotomy for severe pleural adhesions, 1 undergoing concurrent cholecystectomy, and 3 in the non-intubation group converted to intubated anesthesia (1 for significant mediastinal movement and 2 for extensive pleural adhesions). Ultimately, 169 patients were available for analysis, including 104 patients in the intubation group and 65 patients in the non-intubation group. The type of segmentectomy by anatomic location was shown in Supplemental Table 5. Complex segmentectomies accounted for 72.9% and 70.2% of cases in the non-intubated and intubated groups, respectively. The levels of inflammatory cytokines (IL-6, IL-10, HNL), blood gas parameters and 30-day mortality had missing values (Supplemental Table 6). The diagnostic results of multiple imputation showed good convergence and no evidence of systematic bias. After propensity score matching, 43 patients from the intubation group and 4 from the non-intubation group were excluded, yielding a final cohort of 122 patients (61 per group) (Figure 1). All baseline and demographic characteristics were well balanced in the matched cohort (Table 1).

**Table 1.** Baseline characteristics before and after PSM.

	Before PSM				After PSM			
Variable	Non-intubation	Intubation	SMD	*p*	Non-intubation	Intubation	SMD	*p*
	*n* = 65	*n* = 104			*n* = 61	*n* = 61		
Sex (male)	27 (41.5)	46 (44.2)	0.054	0.854	25 (41.0)	27 (44.3)	0.066	0.855
Age, years	54.97 ± 11.23	58.5 ± 10.99	0.318	0.045	56.08 ± 10.07	55.70 ± 11.71	0.035	0.849
BMI, kg/m^2^	23.92 ± 2.82	24.51 ± 3.26	0.196	0.225	24.12 ± 2.77	24.31 ± 3.15	0.063	0.726
FEV_1_ (%pred)	101.40 (89.00, 113.30)	100.30 (88.05, 112.65)	0.037	0.979	103.70 (89.00, 113.40)	100.20 (89.10, 110.00)	0.072	0.924
FEV_1_/FVC (%pred)	105.80 (100.00, 111.00)	102.85 (98.55, 109.33)	0.160	0.205	106.00 (100.30, 111.00)	103.50 (99.00, 109.70)	0.018	0.573
ARISCAT grade			0.138	0.682			0.063	0.423
Low risk	4 (6.2)	5 (4.8)			3 (4.9)	4 (6.6)		
Intermediate risk	35 (53.8)	63 (60.6)			34 (55.7)	33 (54.1)		
High risk	26 (40.0)	36 (34.6)			24 (39.3)	24 (39.3)		
Smoking			0.360	0.097			0.053	0.957
No	54 (83.1)	74 (71.2)			50 (82.0)	49 (80.3)		
Former	5 (7.7)	7 (6.7)			5 (8.2)	5 (8.2)		
Current	6 (9.2)	23 (22.1)			6 (9.8)	7 (11.5)		
ASA physical status			0.163	0.581			0.077	0.913
I	19 (29.2)	23 (22.1)			16 (26.2)	18 (29.5)		
II	33 (50.8)	58 (55.8)			32 (52.5)	30 (49.2)		
III	13 (20.0)	23 (22.1)			13 (21.3)	13 (21.3)		
Charlson comorbidity index	0 (0, 1)	1 (0, 1)	0.264	0.025	0 (0, 1)	0 (0, 1)	0.017	0.399
Hypertension	13 (20.0)	29 (27.9)	0.186	0.332	13 (21.3)	17 (27.9)	0.153	0.528
Cardiovascular disease	7 (10.8)	11 (10.6)	0.006	1.000	7 (11.5)	5 (8.2)	0.110	0.761
Diabetes mellitus	6 (9.2)	14 (13.5)	0.134	0.559	6 (9.8)	6 (9.8)	<0.001	>0.999
Anesthesia duration	155.00 (145.00, 195.00)	160.00 (140.00, 185.00)	0.097	0.819	155.00 (145.00, 191.50)	160.00 (140.00, 195.00)	0.037	0.641
Surgery duration, min	135.00 (117.50, 170.00)	130.00 (108.50, 155.00)	0.182	0.335	130.00 (117.50, 164.50)	130.00 (110.00, 165.00)	0.044	0.782
Segmentectomy			0.081	0.736			0.037	>0.999
Simple	17 (26.2)	31 (29.8)			17 (27.9)	16 (26.2)		
Complex	48 (73.8)	73 (70.2)			44 (72.1)	45 (73.8)		
Concurrent wedge resection	9 (13.8)	13 (12.5)	0.019	0.800	9 (14.8)	9 (14.8)	<0.001	>0.999
Pleural adhesiolysis	14 (21.5)	19 (18.3)	0.040	0.602	13 (21.3)	12 (19.7)	0.020	0.823
Lymph nodes dissection or sampling	64 (98.5)	101 (97.1)	0.043	0.968	61 (100.0)	59 (96.7)	0.129	0.476
Total fluid, mL·kg^–1^·h^–1^	3.75 (3.45, 4.04)	3.74 (3.52, 3.89)	0.023	0.974	3.72 (3.42, 4.00)	3.72 (3.56, 3.89)	0.064	0.525
Pathology			0.070	0.827			0.047	>0.999
Benign	9 (13.8)	17 (16.3)			8 (13.1)	9 (14.8)		
Malignant	56 (86.2)	87 (83.7)			53 (86.9)	52 (85.2)		

Data are presented as *n* (%), mean ± standard deviation, or median (IQR).

PSM, Propensity score matching; SMD, standardized mean differences; BMI, body mass index; FEV_1_, forced expiratory volume in 1 s; FVC, forced vital capacity; ARISCAT, Assess Respiratory Risk in Surgical Patients in Catalonia; ASA, American Society of Anesthesiologists Physical Status Classification; IQR, interquartile range.

### Outcomes

In the matched cohort, the incidence of PPCs was significantly lower in the non-intubation group compared to the intubation group (16.4% vs. 37.7%; RR, 0.75; 95% confidence interval [CI], 0.60 to 0.93; *p* = 0.008). Among all PPCs, only the reduction in respiratory infections was significant (9.8% vs. 29.5%; RR, 0.78; 95% CI, 0.65 to 0.94; *p* = 0.006). Results in the full cohort and ITTanalysis were like those in the matched cohort (Table 2, Supplemental Table 7). In addition, there were no significant interactions between intubation strategy and predefined subgroup variables on the primary outcome (Supplemental Figure 1).

**Table 2.** Primary outcomes in patients before and after PSM.

	Full cohort				Matching cohort			
	Non-intubation	Intubation			Non-intubation	Intubation		
Variable	*n* = 65	*n* = 104	RR (95% CI)	*p*	*n* = 61	*n* = 61	RR (95% CI)	*p*
PPCs within 7 days	11 (16.9)	41 (39.4)	0.73 (0.60, 0.88)	0.002	10 (16.4)	23 (37.7)	0.75 (0.60, 0.93)	0.008
Respiratory infection	6 (9.2)	34 (32.7)	0.74 (0.64, 0.87)	<0.001	6 (9.8)	18 (29.5)	0.78 (0.65, 0.94)	0.006
Atelectasis	5 (7.7)	8 (7.7)	1.00 (0.91, 1.09)	>0.999	4 (6.6)	5 (8.2)	0.98 (0.89, 1.09)	>0.999
Pleural effusion	2 (3.1)	7 (6.7)	0.96 (0.90, 1.03)	0.498	2 (3.3)	4 (6.6)	0.97 (0.89, 1.05)	0.675
Pneumothorax	0	0	NA	NA	0	0	NA	NA
Respiratory failure	0	0	NA	NA	0	0	NA	NA
Bronchospasm	0	0	NA	NA	0	0	NA	NA
Aspiration pneumonitis	0	0	NA	NA	0	0	NA	NA

Data are presented as *n* (%).

PSM, propensity score matching; PPCs, postoperative pulmonary complications; RR, relative risk; CI, confidence interval; NA, not applicable.

Among secondary outcomes, the severity score of PPCs was significantly reduced in the non-intubation group compared with that in the intubation group in the matched cohort (*p* = 0.030). The length of hospital stay after surgery was shorter in non-intubation group (2 [2–3] days vs. 3 [2–3] days; median differences, 0.00; 95% CI, −1.00 to 0.00; *p* = 0.003). The drainage volume was less in the non-intubation group compared with intubation group (80.00 [50.00, 130.00] mL vs. 150.00 [50.00, 280.00] mL; median differences, −45.00; 95% CI, −90.00 to −5.00; *p* = 0.023). The incidence of extrapulmonary complications and postoperative pulmonary air leakage, the drainage duration, were similar between the two groups. Additionally, we found a lower level of WBC in non-intubation group (9.33 [7.49, 12.51] × 10^9^/L vs. 11.23 [9.19, 13.04] × 10^9^/L; median differences, −1.38; 95% CI, −2.46 to −0.04; *p* = 0.044) (Table 3).

**Table 3.** Second outcomes in patients before and after PSM.

	Full cohort				Matching cohort			
	Non-intubation	Intubation	Differences/RR (95% CI)	*p*	Non-intubation	Intubation	Differences/RR (95% CI)	*p*
Variable	*n* = 65	*n* = 104			*n* = 61	*n* = 61		
PPCs severity grade			NA	0.004*			NA	0.030*
0	25 (38.5)	30 (28.8)			25 (41.0)	20 (32.8)		
1	26 (40.0)	28 (26.9)			23 (37.7)	16 (26.2)		
2	8 (12.3)	7 (6.7)			7 (11.5)	3 (4.9)		
3	6 (9.2)	39 (37.5)			6 (9.8)	22 (36.1)		
>3	0	0			0	0		
Extrapulmonary complications within 7 days	14 (21.5)	32 (30.8)	0.88 (0.74, 1.06)	0.190	14 (23.0)	18 (29.5)	0.92 (0.74, 1.13)	0.410
Postoperative pulmonary air leakage	2 (3.1)	4 (3.8)	0.99 (0.94, 1.05)	>0.999	2 (3.3)	3 (4.9)	0.98 (0.91, 1.06)	>0.999
Drainage duration, day	1.00 (1.00, 2.00)	2.00 (1.00, 2.00)	0.00 (0.00, 0.00)	0.011	1.00 (1.00, 2.00)	2.00 (1.00, 2.00)	0.00 (0.00, 1.00)	0.067
Drainage volume, mL	80.00 (50.00, 130.00)	125.00 (50.00, 230.00)	−40.00 (–75.00, 10.00)	0.010	80.00 (50.00, 130.00)	150.00 (50.00, 280.00)	−45.00 (–90.00, −5.00)	0.023
Postoperative hospital stay, day	2.00 (2.00, 3.00)	3.00 (2.00, 3.00)	0.00 (–1.00, 0.00)	0.005	2.00 (2.00, 3.00)	3.00 (2.00, 3.00)	0.00 (–1.00, 0.00)	0.003
WBC	9.33 (7.54, 12.36)	11.09 (9.00,13.44)	−1.28 (–2.27, −0.11)	0.033	9.33 (7.49, 12.51)	11.23 (9.19, 13.04)	−1.38 (–2.46, −0.04)	0.044
CRP	68.20 (36.40, 111.30)	74.35 (45.00, 134.15)	−7.30 (–24.20, 8.90)	0.340	72.80 (34.05, 111.30)	71.50 (39.35, 123.45)	−1.90 (–20.10, 17.80)	0.832
HNL	92.29 (56.07, 127.31)	101.42 (63.02, 146.76)	−8.98 (–26.21, 6.24)	0.232	91.21 (51.83, 126.32)	103.97 (68.67, 143.41)	−16.58 (–36.67, 1.36)	0.077
IL-6	60.66 (30.38, 130.38)	63.97 (36.48, 117.55)	−2.53 (–16.59, 13.27)	0.686	60.66 (29.28, 133.33)	56.00 (38.88, 87.74)	−0.48 (–13.82, 21.13)	0.967
IL-10	4.17 (2.98, 7.72)	5.21 (3.61, 8.04)	−0.58 (–1.41, 0.20)	0.149	4.28 (3.26, 8.27)	4.92 (3.51, 7.36)	−0.07 (–0.96, 0.77)	0.790

Data are presented as *n* (%) or median (IQR).

PSM, propensity score matching; PPCs, postoperative pulmonary complications; ICU, intensive care unit; WBC, white blood cell; CRP, C-reactive protein; HNL, human neutrophil lipocalin; IL, interleukin; RR, relative risk; IQR, interquartile range; CI, confidence interval; NA, not applicable.

Regarding safety endpoints, the non‑intubation group had significantly greater severity of intraoperative hypercapnia and higher grades of mediastinal movement than the intubation group in both the ITT and matched ITT cohorts (both *p* < 0.001). In contrast, no significant differences were observed between the two groups in intraoperative hypoxemia, cough interference, R0 resection rate, lymph nodes yield, blood transfusion, intraoperative air leak, and conversion to intubated anesthesia or thoracotomy. Serious adverse events, including unplanned ICU admission, reoperation, in‑hospital mortality and 30‑day unplanned readmission were similar between the two groups (all *p* > 0.05). Eighteen patients were lost to 30-day mortality follow-up. No deaths were recorded in either group among patients with complete follow-up (Table 4).

**Table 4.** Safety outcomes of the ITT analysis before and after PSM.

	Full cohort				Matching cohort			
	Non-intubation	Intubation	Differences/RR (95% CI)		Non-intubation	Intubation	Differences/RR (95% CI)	
Variable	*n* = 70	*n* = 104		*p*	*n* = 65	*n* = 65		*p*
Hypoxemia^a^	2 (2.9)	8 (7.7)	0.95 (0.89, 1.02)	0.312	2 (3.1)	6 (9.2)	0.94 (0.86, 1.02)	0.274
Hypercapnia^b^			NA	<0.001*			NA	<0.001*
Mild	5 (7.1)	23 (22.1)			5 (7.7)	15 (23.1)		
Moderate	60 (85.7)	8 (7.7)			57 (87.7)	4 (6.2)		
Severe	2 (2.9)	0			0	0		
Mediastinal movement			NA	<0.001*			NA	<0.001*
Mild	45 (64.3)	0			41 (63.1)	0		
Moderate	20 (28.6)	0			19 (29.2)	0		
Severe	5 (7.1)	0			5 (7.7)	0		
Cough interference	6 (8.6)	4 (3.8)	2.23 (0.65, 7.61)	0.327	5 (7.7)	2 (3.1)	2.50 (0.50, 12.42)	0.437
R0 resection rate	70 (100.0)	104 (100.0)	NA	NA	65 (100.0)	65 (100.0)	NA	NA
Number of lymph nodes sampled or dissected	6.00 (3.75, 9.00)	6.00 (4.00, 7.00)	0.00 (–1.00, 1.00)	0.853	6.00 (4.00, 9.00)	5.00 (4.00, 7.00)	0.00 (–1.00, 1.00)	0.631
Estimated blood loss, mL	40.0 (30.0, 50.0)	40.0 (30.0, 50.0)	0.0 (0.0, 10.0)	0.089	40.0 (30.0, 50.0)	40.0 (30.0, 50.0)	0.0 (0.0, 10.0)	0.149
Blood transfusion	0	0	NA	NA	0	0	NA	NA
Intraoperative air leak	27 (38.6)	37 (35.6)	1.05 (0.83, 1.33)	0.688	26 (40.0)	25 (38.5)	1.03 (0.78, 1.35)	0.857
Conversion to intubated anesthesia	3 (4.3)	0	NA	0.063	3 (4.6)	0	NA	0.244
Conversion to thoracotomy	2 (2.9)	0	NA	0.160	2 (3.1)	0	NA	0.496
Unplanned ICU admission	0	2 (1.9)	NA	0.516	0	1 (1.5)	NA	>0.999
In-hospital mortality	0	0	NA	NA	0	0	NA	NA
Reoperation	0	0	NA	NA	0	0	NA	NA
30-day unplanned readmission	0	2 (1.9)	NA	0.516	0	0	NA	NA
30-day mortality^c^	0	0	NA	NA	0	0	NA	NA

Data are presented as *n* (%) or median (IQR).

PSM, propensity score matching; ITT, intention-to-treat; ICU, intensive care unit; PaCO_2_, partial pressure of carbon dioxide in arterial blood; RR, relative risk; CI, confidence interval; NA, not applicable.

^a^ Hypoxemia was defined as SpO_2_ < 92 when the FiO_2_ has been set at 100%.

^b^ Hypercapnia was stratified into mild (PaCO_2_ 45–50 mmHg), moderate (50–70 mmHg) and severe (>70 mmHg) grades according to arterial carbon dioxide tension.

^c^ 30-day mortality data only include patients with complete 30-day follow-up (*n* = 156); 18 patients were lost to follow-up and excluded from this calculation.

* *U* test.

### Sensitivity analysis

In a pre-specified sensitivity analysis using multivariate logistic regression within the ITT cohort, non-intubation was identified as an independent protective factor against PPCs (adjusted OR, 0.29; 95% CI, 0.13–0.61; *p* = 0.001), alongside a significant overall effect of ARISCAT grade (*p* = 0.017) (Table 5). Moreover, the similar results were observed after reassessing PPCs using both the MGS and StEP definitions (Supplemental Table 8).

**Table 5.** Univariate and multivariate logistic regression analysis of PPCs in the ITT cohort.

	Univariate logistic analysis*		Multivariate logistic analysis	
	OR (95% CI)	*p*	Adjusted OR (95% CI)	*p*
Non-intubation (ref: intubation)	0.32 (0.15, 0.66)	0.002	0.29 (0.13, 0.61)	0.001
ARISCAT grade (ref: low risk)		0.031		0.017
Intermediate risk	2.63 (0.31, 22.09)	0.373	2.54 (0.29, 22.00)	0.398
High risk	5.84 (0.69, 49.48)	0.106	6.45 (0.73, 56.77)	0.093

PPCs, postoperative pulmonary complications; ARISCAT, Assess Respiratory Risk in Surgical Patients in Catalonia; OR, odds ratio; CI, confidence interval.

* Variables with *p* < 0.2 in univariate analysis were included in multivariate regression analysis.

## Discussion

In patients undergoing VATS segmentectomy, we found that non-intubated anesthesia was associated with significantly decreased incidence and severity of PPCs within 7 days after surgery. Among the PPCs, the reduction of respiratory infections was most significant. The association between non-intubation anesthesia and decreased risk of PPCs persisted after reevaluating PPCs by different definitions and multiple logistic regression analysis. Under our study conditions, no differences between non-intubated anesthesia and intubated anesthesia were noted in postoperative pulmonary air leakage, extrapulmonary complications, admission to ICU and in-hospital mortality. Moreover, the length of postoperative hospital stay was shorter in non-intubation group than intubation group.

Currently, there are few studies investigating the relationship between non-intubated anesthesia and PPCs following VATS segmentectomy. Inconsistent with our findings, previous studies have not demonstrated a decreased incidence of PPCs in patients undergoing non-intubated anesthesia VATS segmentectomy [28,29]. Possible reasons for the discrepancy include differences in PPCs definitions, intraoperative management, proportion of simple and complex segmentectomy, or patient characteristics. We hypothesized that the reduction of respiratory infections in non-intubation group may be attributed to several reasons. Firstly, the process of intubation may carry exogenous microorganisms from the mouth or surrounding environment into the trachea, leading to respiratory infection. Secondly, the invasion of intubation could damage the integrity of the tracheal mucosa, increasing the susceptibility of the airway to microorganisms and triggered an inflammatory response [30]. Thirdly, ventilation-associated lung injury is also a common risk for PPCs. Mechanical ventilation increased vascular endothelium permeability, pulmonary oedema and hypoxia, which induced the inflammatory cell infiltration and led to lung injury [8]. Non-intubated anesthesia preserved spontaneous breathing and avoided intubated mechanical ventilation during surgery, which may be beneficial in reducing the risk of postoperative respiratory infections. An animal study showed lower TNF-α level and pathological injury score in spontaneous breathing group than OLV group [31]. Fourthly, anesthetics may also give rise to PPCs. The POPULAR study showed that neuromuscular blocking drugs was associated with an increased risk of PPCs [10]. Given that non-intubated anesthesia significantly reduces the amounts of anesthetic agents such as opioids and muscle paralyzing agents, it may improve the PPCs induced by anesthetics. Additionally, no complications such as pneumothorax, respiratory failure, bronchospasm, and aspiration pneumonitis were observed in our cohort. This may be attributable to the low incidence rates of these pulmonary complications, combined with our insufficient sample size, the highly selective nature of the patient population, and a follow-up period limited to postoperative day 7, which may have been inadequate to capture such events.

In this study, we conducted a multidimensional evaluation of postoperative pulmonary outcomes using a validated PPCs severity grading system and identified milder PPCs severity in patients receiving non-intubated anesthesia. This between-group difference exhibits an observational correlation with perioperative immune and inflammatory biomarker patterns seen in the non-intubated cohort. In line with earlier studies, the non-intubated anesthesia cohort exhibited lower postoperative leukocyte counts, alongside reduced IL-6 and higher IL-2 and IL-17A concentrations [20,32,33]. Importantly, all such immune and inflammatory shifts are interpreted solely as exploratory observational endpoints, rather than conclusive mechanistic proof. This cautious interpretation accounts for the constraints of single-time-point biomarker sampling and the numerous overlapping perioperative variables capable of altering systemic inflammatory status. Consistent with previous studies [28,29], the incidence of severe adverse events including intraoperative air leak, extrapulmonary complications, unplanned ICU admission, reoperation, in-hospital mortality, and 30-day mortality was comparable between the two groups, further supporting the safety and feasibility of non-intubated anesthesia for thoracoscopic segmentectomy. Furthermore, prior studies mainly focused on simple segmentectomies, while complex segmentectomies accounted for up to 70% of our cohort. This finding suggests non-intubated anesthesia is safe and feasible for both simple and complex pulmonary segmental resections. Consistent with earlier thoracoscopic lung surgery research [34,35], our non-intubation group achieved significantly shorter postoperative hospital stays and reduced chest drainage volume relative to the intubation group following VATS segmentectomy, which highlights the capacity of non-intubated anesthesia to accelerate postoperative recovery. However, these findings should not be interpreted as evidence of no clinical effect, given the limited sample size and statistical power.

Mediastinal movement represents an inevitable and critical challenge during non-intubated thoracoscopic surgery. In our cohort, mild and moderate mediastinal movement predominated; such disturbance slightly hindered surgical manipulation without compromising procedural integrity. The 100% R0 resection rate and comparable lymph node retrieval across both groups further verify that non-intubated anesthesia does not compromise oncologic surgical quality. In fact, existing evidence suggests that lung cancer patients receiving non-intubated anesthesia may achieve superior long-term outcomes [36]. Beyond mediastinal movement, hypercapnia is another challenge associated with non‑intubated anesthesia, as has been well described in prior literature [37,38]. A plausible explanation is that during spontaneous breathing, patients have a lower tidal volume (approximately 300 mL) compared to mechanical ventilation, which can satisfy the oxygenation requirements during the procedure but also leads to carbon dioxide retention. Despite the occurrence of moderate intraoperative hypercapnia in the non-intubation group, hypercapnia-related adverse events were not observed. This may be attributed to the strict exclusion criteria applied to the non-intubated population, which excluded patients at high risk such as those with obesity, difficult airways, or severe cardio-pulmonary compromise. Consequently, the generalizability of the results presented herein should be interpreted with caution. Interestingly, recent studies have found that mild-moderate hypercapnia played an anti-inflammatory role in mechanical ventilation lung injury models and improved diaphragm function, indicating its potential for lung protection during surgery [39,40].

## Study limitations

Several limitations of this study warrant consideration. First, as a retrospective observational design, it can only establish associations, not causality. Second, the retrospective nature of clinical trial registration also limits interpretability. Third, despite 1:1 propensity score matching, selection bias cannot be fully excluded due to the lack of randomization—patients receiving non-intubated anesthesia inherently had more favorable baseline characteristics, and treatment allocation was influenced by anesthesiologist preference and experience. Therefore, causal inferences regarding the protective effect of non-intubated anesthesia on PPCs should be drawn with caution. Fourth, non-significant associations for surgical variables could stem from limited sample size, uniform surgeon experience, and individualized anesthetic preferences. Unmeasured or subtle differences in surgical complexity and intraoperative manipulation may still introduce residual confounding and limit the generalizability of the conclusions. Fifth, PPCs were assessed only within the first 7 postoperative days or until discharge, which may underestimate delayed complications such as late pneumonia, readmission for respiratory failure, or prolonged atelectasis. Moreover, a lack of long-term survival follow-up and incomplete capture of 30-day mortality data, particularly in the malignancy subgroup, precludes any conclusion on the oncological impact of this anesthetic technique. Finally, as a single-center study, the generalizability of our findings is inherently limited. Future prospective multicenter studies with extended postoperative surveillance are needed to validate whether the benefits of non-intubated anesthesia extend beyond the acute phase.

## Conclusion

For carefully selected patients undergoing VATS segmentectomy managed by an experienced multidisciplinary team, non-intubated anesthesia represents a feasible alternative to conventional intubated anesthesia. In this single-center retrospective cohort, non-intubated anesthesia was correlated with a lower incidence and attenuated severity of PPCs within the first 7 days following thoracoscopic segmentectomy.

## Supplementary Material

Supplemental Material

STROBE Statement.docx

## Data Availability

The data supporting the findings of this study are available upon reasonable request from the corresponding author, Ning Li (lining252252@126.com).
