# Supplementary material for: Effects of non-intubated anesthesia on postoperative pulmonary complications after thoracoscopic segmentectomy: a propensity score-matched analysis
Source: Ann Med. 2026 Aug 26;58(1):2721209. doi: 10.1080/07853890.2026.2721209 (PMC13520849; doi:10.1080/07853890.2026.2721209)
Supplement: Supplementary file 2 [file IANN_A_2721209_SM5405.docx]

**Supplemental Tables**

Supplemental Table 1. EPCO definition of postoperative pulmonary complications.

Supplemental Table 2. Definition of postoperative pulmonary complications severity grade.

Supplemental Table 3. Definition of Clavien-Dindo classification.

Supplemental Table 4. ARISCAT risk score and grade

Supplemental Table 5. Type of segmentectomy by anatomic location in the ITT cohort.

Supplemental Table 6. Number of missing cases in the ITT cohort.

Supplemental Table 7. Primary outcomes of the ITT analysis before and after PSM.

Supplemental Table 8. Different definitions of postoperative pulmonary complications for sensitivity analysis.

Supplemental Figure 1. Forest plot in predefined subgroup. BMI, body mass index; ARISCAT, Assess Respiratory Risk in Surgical Patients in Catalonia; OR, odds ratio; CI, confidence interval.

**Supplemental Table 1. EPCO definition of postoperative pulmonary complications.**

| Complications | Definition |
| --- | --- |
| Respiratory infection | Patient has received antibiotics for a suspected respiratory infection and met one or more of the following criteria: new or changed sputum, new or changed lung opacities, fever, white blood cell count > 12×10^9^ |
| Pneumothorax | Air in the pleural space with no vascular bed surrounding the visceral pleura |
| Atelectasis | Lung opacification with a shift of the mediastinum, hilum or hemidiaphragm toward the affected area, and compensatory over-inflation in the adjacent non-atelectatic lung |
| Pleural effusion | Chest radiograph demonstrating blunting of the costophrenic angle, loss of sharp silhouette of the ipsilateral hemidiaphragm in upright position, evidence of displacement of adjacent anatomical structures or (in supine position) a hazy opacity in one hemithorax with preserved vascular shadows |
| Bronchospasm | Newly detected expiratory wheezing treated with bronchodilators |
| Aspiration pneumonitis | Acute lung injury after the inhalation of regurgitated gastric contents |
| Respiratory failure | Postoperative PaO_2_ < 8 kPa (60 mmHg) on room air, a PaO_2_:FiO_2_ ratio <40 kPa (300 mmHg) or arterial oxyhaemoglobin saturation measured with pulse oximetry < 90% and requiring oxygen therapy |

EPCO, European Perioperative Clinical Outcome; PaO_2_, partial pressure of arterial oxygen; FiO_2_, fraction of inspiration oxygen.

**Supplemental Table 2.** **Definition of postoperative pulmonary complications severity grade.**

| Grade | Definition |
| --- | --- |
| Grade 0 | -No symptoms or signals |
| Grade 1 | -Cough, dry |
|  | -Microatelectasis: abnormal lung findings and temperature > 37.5°C without other documented cause; normal chest radiograph |
|  | -Dyspnea, not due to other documented cause |
| Grade 2 | -Cough, productive, not due to other documented cause |
|  | -Bronchospasm: new wheezing or preexistent wheezing resulting in a change in therapy |
|  | -Hypoxemia: SpO_2_ < 90% at room air |
|  | -Atelectasis: gross radiological confirmation (concordance of 2 independent experts) plus either temperature > 37.5°C or abnormal lung findings |
|  | -Hypercarbia (PaCO_2_ > 50mmHg), requiring treatment |
| Grade 3 | -Pleural effusion, resulting in thoracentesis |
|  | -Pneumonia: radiological evidence (concordance of 2 independent experts) plus clinical symptoms (two of the following: leucocytosis or leucopenia, abnormal temperature, purulent secretions), plus either a pathological organism (by Gram stain or culture) or a required change in antibiotics |
|  | -Pneumothorax |
|  | -Noninvasive ventilation, strictly applied to those with all of the following: a) SpO_2_ ≤ 92% under supplemental oxygen; b) need of supplemental oxygen > 5L min^-1^; and respiratory rate ≥ 30 bpm |
|  | -Reintubation postoperative or intubation, period of ventilator dependence does not exceed 48 hours |
| Grade 4 | -Ventilatory failure: postoperative ventilator dependence exceeding 48 hours, or reintubation with subsequent period of ventilator dependence exceeding 48 hours |
| Grade 5 | -Death |

SpO_2_, pulse oxygen saturation; PaCO_2_, partial pressure of arterial carbon dioxide.

**Supplemental Table 3. Definition of Clavien-Dindo classification.**

| Grade | Definition |
| --- | --- |
| I | -Any deviation from the normal postoperative course without the need for pharmacological treatment or surgical, endoscopic, or radiological interventions. |
|  | -Allowed therapeutic regimens include drugs such as antiemetics, antipyretics, analgesics, diuretics, electrolytes, and physiotherapy. This grade also includes wound infections opened at the bedside. |
| II | -Requirement for pharmacological treatment with drugs other than those allowed for grade I complications. |
|  | -Blood transfusions and total parenteral nutrition are also included. |
| IIIa | -Requirement for surgical, endoscopic or radiological intervention not under general anesthesia. |
| IIIb | -Requirement for surgical, endoscopic or radiological intervention under general anesthesia. |
| IVa | -Life-threatening complications (including CNS complications) requiring IC/ICU management. |
|  | -Single organ dysfunction (including dialysis). |
| IVb | -Life-threatening com- plications (including CNS complications)^*^ requiring IC/ICU management. |
|  | -Multiple organ dysfunction. |
| V | -Death of the patient. |

CNS, central nervous system; IC, Intermediate care; ICU, intensive care unit.

^*^Brain hemorrhage, ischemic stroke, subarachnoidal bleeding, but excluding transient ischemic attacks.

**Supplemental Table 4. ARISCAT risk score and grade**

| Risk factor | Score |
| --- | --- |
| Age (years) |  |
| ≤ 50 | 0 |
| 51-80 | 3 |
| > 80 | 16 |
| Pre-operative SpO_2_ (%) |  |
| ≥ 96 | 0 |
| 91-95 | 8 |
| ≤ 90 | 24 |
| Respiratory infection in the last month |  |
| No | 0 |
| Yes | 17 |
| Pre-operative anemia (Hb ≤ 10 g dl^-1^) |  |
| No | 0 |
| Yes | 11 |
| Surgical incision |  |
| Peripheral | 0 |
| Upper abdominal | 15 |
| Intrathoracic | 24 |
| Duration of surgery (h) |  |
| ≤ 2 | 0 |
| 2-3 | 16 |
| > 3 | 23 |
| Emergency procedure |  |
| No | 0 |
| Yes | 8 |

ARISCAT, Assess Respiratory Risk in Surgical Patients in Catalonia. SpO_2_, pulse oxygen saturation; Hb, hemoglobin. Three levels of risk were indicated by the following cutoffs: < 26 points, low risk; 26–44 points, moderate risk; and ≥ 45 points, high risk.

**Supplemental Table 5**. **Type of segmentectomy by anatomic location in the ITT cohort.**

| Non-intubation (n=70) | | | |  | Intubation (n=104) | | | |
| --- | --- | --- | --- | --- | --- | --- | --- | --- |
| Complex (n=51) | | Simple (n=19) | |  | Complex (n=73) | | Simple (n=31) | |
| Left | 18 | Left | 12 |  | Left | 30 | Left | 19 |
| S3 | 5 | S6 | 4 |  | S3 | 5 | S6 | 7 |
| S3b+c | 1 | S4+5 | 2 |  | S8 | 1 | S4+5 | 4 |
| S1+2 | 8 | S1+2+3 | 4 |  | S10 | 2 | S1+2+3 | 6 |
| S1+2c+3a | 1 | S7+8+9+10 | 2 |  | S1+2 | 11 | S7+8+9+10 | 2 |
| S8+9 | 1 |  |  |  | S1+2c+S4a | 1 |  |  |
| S8+9+10 | 1 |  |  |  | S3+4b | 1 |  |  |
| S9+10 | 1 |  |  |  | S8+9 | 4 |  |  |
|  |  |  |  |  | S8a+S9a | 1 |  |  |
|  |  |  |  |  | S8a+9 | 1 |  |  |
|  |  |  |  |  | S8+9+* | 1 |  |  |
|  |  |  |  |  | S8+9+10 | 2 |  |  |
| Right | 33 | Right | 7 |  | Right | 43 | Right | 12 |
| S1 | 5 | S6 | 5 |  | S1 | 5 | S6 | 10 |
| S2 | 6 | S7+8+9+10 | 2 |  | S2 | 10 | S7+8+9+10 | 2 |
| S3 | 4 |  |  |  | S3 | 7 |  |  |
| S8 | 5 |  |  |  | S8 | 1 |  |  |
| S10 | 1 |  |  |  | S10 | 2 |  |  |
| S1+2 | 2 |  |  |  | S1+2 | 4 |  |  |
| S1a+2a | 1 |  |  |  | S1a+2 | 1 |  |  |
| S1+2, S4a | 1 |  |  |  | S1+2b | 2 |  |  |
| S1+S3 | 1 |  |  |  | S1+3 | 4 |  |  |
| S1+S4b | 1 |  |  |  | S1b+3b | 1 |  |  |
| S1+2b+3a | 1 |  |  |  | S3b | 1 |  |  |
| S7+8b | 1 |  |  |  | S7+8b | 1 |  |  |
| S8+9 | 2 |  |  |  | S8+9+10 | 1 |  |  |
| S9+10 | 1 |  |  |  | S8+9, S6b | 1 |  |  |
| S6+* | 1 |  |  |  | S8+9, S6b+c | 1 |  |  |
|  |  |  |  |  | S9+10 | 1 |  |  |

Data are presented as number. ITT, intention-to-treat;

**Supplemental Table 6. Number of missing cases in the ITT cohort.**

|  | Non-intubation | Intubation | Total |
| --- | --- | --- | --- |
| Missing data | n=70 | n=104 | n=174 |
| HNL | 12 (17.1) | 19 (18.3) | 31 (17.8) |
| IL-6 | 6 (8.6) | 12 (11.5) | 18 (10.3) |
| IL-10 | 11 (15.7) | 19 (18.3) | 30 (17.2) |
| blood gas parameters | 4 (5.7) | 9 (8.7) | 13 (7.5) |
| 30-day mortality | 5 (7.1) | 13 (12.5) | 18 (10.3) |

Data are presented as n (%). ITT, intention-to-treat; HNL, human neutrophil lipocalin; IL, interleukin.

**Supplemental Table 7. Primary outcomes of the ITT analysis before and after PSM.**

|  | Full cohort | | | |  |  | Matching cohort | | | |
| --- | --- | --- | --- | --- | --- | --- | --- | --- | --- | --- |
|  | Non-intubation | Intubation |  |  |  |  | Non-intubation | Intubation |  |  |
| Variable | n=70 | n=104 | RR (95% CI) | *P* |  |  | n=65 | n=65 | RR (95% CI) | *P* |
| Postoperative pulmonary complications | 12 (17.1) | 41 (39.4) | 0.73 (0.61, 0.88) | 0.002 |  |  | 10 (15.4) | 27 (41.5) | 0.69 (0.55, 0.87) | 0.001 |
| Respiratory infection | 6 (8.6) | 34 (32.7) | 0.74 (0.63, 0.86) | <0.001 |  |  | 6 (9.2) | 21 (32.3) | 0.75 (0.62, 0.90) | 0.001 |
| Atelectasis | 6 (8.6) | 8 (7.7) | 1.01 (0.92, 1.11) | 0.834 |  |  | 4 (6.2) | 6 (9.2) | 0.97 (0.88, 1.07) | 0.510 |
| Pleural effusion | 2 (2.9) | 7 (6.7) | 0.96 (0.90, 1.03) | 0.434 |  |  | 2 (3.1) | 5 (7.7) | 0.95 (0.88, 1.03) | 0.437 |
| Pneumothorax | 0 | 0 | NA | NA |  |  | 0 | 0 | NA | NA |
| Respiratory failure | 0 | 0 | NA | NA |  |  | 0 | 0 | NA | NA |
| Bronchospasm | 0 | 0 | NA | NA |  |  | 0 | 0 | NA | NA |
| Aspiration pneumonitis | 0 | 0 | NA | NA |  |  | 0 | 0 | NA | NA |

Data are presented as n (%). PSM, propensity score matching; ITT, intention-to-treat; RR, relative risk; CI, confidence interval; NA, not applicable.

**Supplemental Table 8. Different definitions of postoperative pulmonary complications for sensitivity analysis.**

|  | Full cohort | | | |  | Matching cohort | | | |
| --- | --- | --- | --- | --- | --- | --- | --- | --- | --- |
| Variable | Non-intubation | Intubation | Relative risk (95% CI) | *P* |  | Non-intubation | Intubation | Relative risk  (95% CI) | *P* |
|  | n=65 | n=104 |  |  |  | n=61 | n=61 |  |  |
| MGS PPCs | 4 (6.2) | 26 (25.0) | 0.80 (0.70, 0.91) | 0.002 |  | 4 (6.6) | 13 (21.3) | 0.84 (0.73, 0.98) | 0.019 |
| StEP PPCs | 9 (13.8) | 34 (32.7) | 0.78 (0.66, 0.92) | 0.006 |  | 8 (13.1) | 18 (29.5) | 0.81 (0.67, 0.98) | 0.027 |
| StEP Atelectasis | 5 (7.7) | 8 (7.7) | 1.00 (0.91, 1.09) | 1.000 |  | 4 (6.6) | 5 (8.2) | 0.98 (0.89, 1.09) | 1.000 |
| StEP Pneumonia | 4 (6.2) | 25 (24.0) | 0.81 (0.71, 0.92) | 0.003 |  | 4 (6.6) | 12 (19.7) | 0.86 (0.75, 0.99) | 0.032 |
| StEP Acute Respiratory | 0 | 0 | NA | NA |  | 0 | 0 | NA | NA |
| StEP Pulmonary Aspiration | 0 | 0 | NA | NA |  | 0 | 0 | NA | NA |
| StEP Pleural effusion | 2 (3.1) | 7 (6.7) | 0.96 (0.90, 1.03) | 0.498 |  | 2 (3.3) | 5 (8.2) | 0.95 (0.87, 1.04) | 0.436 |
| StEP Pulmonary embolism | 0 | 0 | NA | NA |  | 0 | 0 | NA | NA |
| StEP Cardiogenic pulmonary oedema | 0 | 0 | NA | NA |  | 0 | 0 | NA | NA |
| StEP Pneumothorax | 0 | 0 | NA | NA |  | 0 | 0 | NA | NA |
| StEP Bronchospasm | 0 | 0 | NA | NA |  | 0 | 0 | NA | NA |

Data are presented as n (%). PPCs: postoperative pulmonary complications; MGS: Melbourne Group Scale; StEP: Standardised end-points in perioperative medicine; CI, confidence interval; NA, not applicable.

Supplemental Figure 1. Forest plot in predefined subgroup. BMI, body mass index; ARISCAT, Assess Respiratory Risk in Surgical Patients in Catalonia; OR, odds ratio; CI, confidence interval.
